# KIR Variation in Iranians Combines High Haplotype and Allotype Diversity With an Abundance of Functional Inhibitory Receptors

**DOI:** 10.3389/fimmu.2020.00556

**Published:** 2020-04-02

**Authors:** Claudia Alicata, Elham Ashouri, Neda Nemat-Gorgani, Lisbeth A. Guethlein, Wesley M. Marin, Sudan Tao, Lorenzo Moretta, Jill A. Hollenbach, John Trowsdale, James A. Traherne, Abbas Ghaderi, Peter Parham, Paul J. Norman

**Affiliations:** ^1^Department of Immunology, IRCCS Bambino Gesù Children's Hospital, Rome, Italy; ^2^Department of Structural Biology, Stanford University School of Medicine, Stanford, CA, United States; ^3^Department of Microbiology and Immunology, Stanford University School of Medicine, Stanford, CA, United States; ^4^Hematology-Oncology and Stem Cell Transplantation Research Center, Tehran University of Medical Sciences, Tehran, Iran; ^5^School of Medicine, Shiraz Institute for Cancer Research, Shiraz University of Medical Sciences, Shiraz, Iran; ^6^Division of Immunology, Department of Pathology, University of Cambridge, Cambridge, United Kingdom; ^7^Department of Neurology, University of California, San Francisco, San Francisco, CA, United States; ^8^Blood Center of Zhejiang Province, Hangzhou, China; ^9^Division of Personalized Medicine, Department of Immunology and Microbiology, University of Colorado, Anschutz Medical Campus, Aurora, CO, United States

**Keywords:** NK cells, KIR, HLA class I, Iranian populations, immune diversity

## Abstract

Natural killer (NK) cells are innate lymphocytes that eliminate infected and transformed cells. They discriminate healthy from diseased tissue through killer cell Ig-like receptor (KIR) recognition of HLA class I ligands. Directly impacting NK cell function, *KIR* polymorphism associates with infection control and multiple autoimmune and pregnancy syndromes. Here we analyze *KIR* diversity of 241 individuals from five groups of Iranians. These five populations represent Baloch, Kurd, and Lur, together comprising 15% of the ethnically diverse Iranian population. We identified 159 *KIR* alleles, including 11 not previously characterized. We also identified 170 centromeric and 94 telomeric haplotypes, and 15 different *KIR* haplotypes carrying either a deletion or duplication encompassing one or more complete *KIR* genes. As expected, comparing our data with those representing major worldwide populations revealed the greatest similarity between Iranians and Europeans. Despite this similarity we observed higher frequencies of KIR3DL1^*^001 in Iran than any other population, and the highest frequency of HLA-B^*^51, a Bw4-containing allotype that acts as a strong educator of KIR3DL1^*^001^+^ NK cells. Compared to Europeans, the Iranians we studied also have a reduced frequency of 3DL1^*^004, which encodes an allotype that is not expressed at the NK cell surface. Concurrent with the resulting high frequency of strong viable interactions between inhibitory KIR and polymorphic HLA class I, the majority of *KIR-A* haplotypes characterized do not express a functional activating receptor. By contrast, the most frequent *KIR-B* haplotype in Iran expresses only one functional inhibitory KIR and the maximum number of activating KIR. This first complete, high-resolution, characterization of the *KIR* locus of Iranians will form a valuable reference for future clinical and population studies.

## Introduction

Natural killer (NK) cells are essential for human immunity to infection and cancer, and for successful reproduction (1, 2). To discriminate diseased from healthy tissue cells, NK cells express an array of inhibiting and activating cell surface receptors (3, 4). Prominent among these receptors are the killer cell immunoglobulin like receptors (KIR), which educate and modulate NK cell function through interaction with HLA class I (5, 6). KIR are highly polymorphic, a genetically-determined variation that directly impacts NK cell function, and susceptibility to disease (7, 8).

The *KIR* locus spans 150–350 kbp of chromosome 19q13.4 (9). The locus is distinguished by structural and sequence diversity of the 13 constituent genes (*KIR2DL1, KIR2DL2/L3, KIR2DL4, KIR2DL5A, KIR2DL5B, KIR2DS1, KIR2DS2, KIR2DS3, KIR2DS4, KIR2DS5, KIR3DL1/S1, KIR3DL2*, and *KIR3DL3*) and two pseudogenes (*KIR2DP1* and *KIR3DP1*) (10, 11). KIR have either two (2D) or three (3D) specificity-determining immunoglobulin-like domains and a long (L) or short (S) tail (12). KIR having a long cytoplasmic tail are inhibitory, whereas those having a short cytoplasmic tail are activating. The one exception is KIR2DL4, which can exhibit inhibitory or activating function (13–15). The *KIR* locus segregates in two main haplotype forms that are maintained in all human populations by balancing selection (16). The *KIR*-*A* haplotypes have a fixed number of predominantly inhibitory receptors, whereas the *KIR-B* haplotypes are characterized by a variable number of both activating and inhibitory receptors. Further distinguishing *KIR-A* and *-B* haplotypes are their characteristic alleles (17). *KIR-A* haplotypes are associated with controlling infectious disease and cancer, but confer susceptibility to reproductive disorders, whereas *KIR-B* haplotypes are associated with protection from reproductive disorders (1, 7). Additionally, haploidentical transplantation therapy for leukemia has an increased success rate when the stem cell donors carry *KIR-B* haplotypes (18, 19).

Polymorphism of *KIR* affects cell surface expression, ligand specificity, ligand binding strength, and intracellular signaling (20–25). All these factors affect the capacity of NK cells to recognize and kill target cells. Because both *KIR* polymorphism and associated diseases are unevenly distributed worldwide, it is critical to fully gauge the genetic diversity of *KIR* in well-defined human populations. Despite this importance, only a few populations have been studied to high resolution, principally due to the complexity of the *KIR* locus. These studies have focused on representative populations of Amerindians (Yucpa) (26), divergent African groups (27–29), Europeans (30, 31), East Asians (Japanese) (32), and Oceanians (Māori) (33). Together, they show how *KIR* allele and haplotype diversity varies dramatically between human populations and highlight the importance of extending *KIR* allele analysis to represent all ethnicities and geographical areas. In this regard, our recent analysis of *HLA* allelic diversity in Iran revealed the highest frequencies of HLA-B^*^51:01 worldwide (34). HLA-B^*^51 contains the Bw4 epitope and interacts KIR3DL1 (35, 36). In the present study we fully characterize *KIR* locus diversity of the same cohort of Iranian individuals.

## Materials and Methods

### Study Population

The *KIR* locus diversity of three indigenous Iranian populations was determined to high-resolution by analyzing genomic DNA from 241 healthy unrelated donors (34). The populations studied represent Baloch, Kurd, and Lur, together comprising 15% (12 million individuals) of the ethnically diverse Iranian population. Studied were 160 Lurs and 48 Kurds, from the Zagros Mountains at the west of Iran, and 33 Baloch from the southeast of Iran. The Lur population included 64 individuals from the city of Khoramabad in the province of Lorestan, 81 from Yasuj in the province of Kohgiluyeh and Boyer-Ahmad, and 15 from Lordegan in the province of Chaharmahal and Bakhtiari. The samples from Kurds were collected from the city of Sanandaj. With the exception of the Baloch, the *HLA class I* alleles were described previously (34). The ancestors of every individual studied had been part of their respective population for at least two generations. Sample collection was approved by the Medical Research Ethics Committee of Shiraz University of Medical Sciences. All participants gave informed consent. Banked, de-identified samples were used for this study.

### Library Preparation and Enrichment

Genomic DNA was prepared by shearing with sonication and the *KIR* genomic region was enriched from the genomic libraries using a pool of oligonucleotide probes as described (37). The enriched fragments were subjected to paired-end sequencing using Illumina's MiSeq instrument and V3 sequencing chemistry (Illumina, La Jolla CA). The sequencing read length was 2 × 300 bp.

### Next Generation Sequence Data Processing and Analysis

Sequence reads specific to the *KIR* region were identified and harvested using Bowtie 2 (38). KIR genotyping was performed using the Pushing Immunogenetics to the Next Generation pipeline (37). This pipeline generates a high-resolution *KIR* gene content and allele level genotype. It can also identify previously unreported single nucleotide polymorphisms (SNPs) and recombinant alleles. Novel allele sequences were analyzed by visual inspection: reads specific to the relevant gene were isolated by bioinformatics filtering, aligned to the closest reference allele using MIRA 4.0.2 (39), and inspected using Gap4 of the Staden package (40) or Integrative Genomics Viewer (41).

### Allele and Haplotype Frequencies

Allele frequencies were calculated by direct counting. The composition and frequencies of *KIR* haplotypes were determined at the allelic level using PHASE 2.1 (42). The following parameters for PHASE 2.1 were used: –f1, –x5, and –d1. Because of the high rate of recombination between *KIR3DP1* and *KIR2DL4* (9), we performed two separate PHASE runs, one for the *KIR* genes of the centromeric region and one for telomeric *KIR* genes. Genes analyzed were *KIR3DL3, 2DS2, 2DL2/3, 2DL5A* and *B, 2DS3/5, 2DP1, 2DL1, 2DL4, 3DL1/S1, 2DS1, 2DS4*, and *3DL2*. For each haplotype we calculated the frequency by direct counting.

### Statistical Analysis

Hardy-Weinberg equilibrium proportions was examined using Fisher's exact test. Differences in frequency amongst populations were tested using χ^2^ with Bonferroni correction for the number of alleles at the respective locus. Fisher's and χ^2^ test as well as Mann-Whitney *U*-test were implemented using GraphPad Prism 7.05.

### Genetic Distance

The genetic distance between Iranians and other populations was calculated using the Cavalli-Sforza model (43), implemented in GENETIX 4.05 (https://kimura.univ-montp2.fr/genetix/). The populations used were Japanese (*N* = 115) (32), Yucpa Amerindians (*N* = 61) (26), Ghanaians from West Africa (*N* = 131) (27), Māori from New Zealand (*N* = 49) (33), Europeans (*N* = 378) (44), and Khomani from South Africa (*N* = 79) (28).

## Results

**Figure 1:**
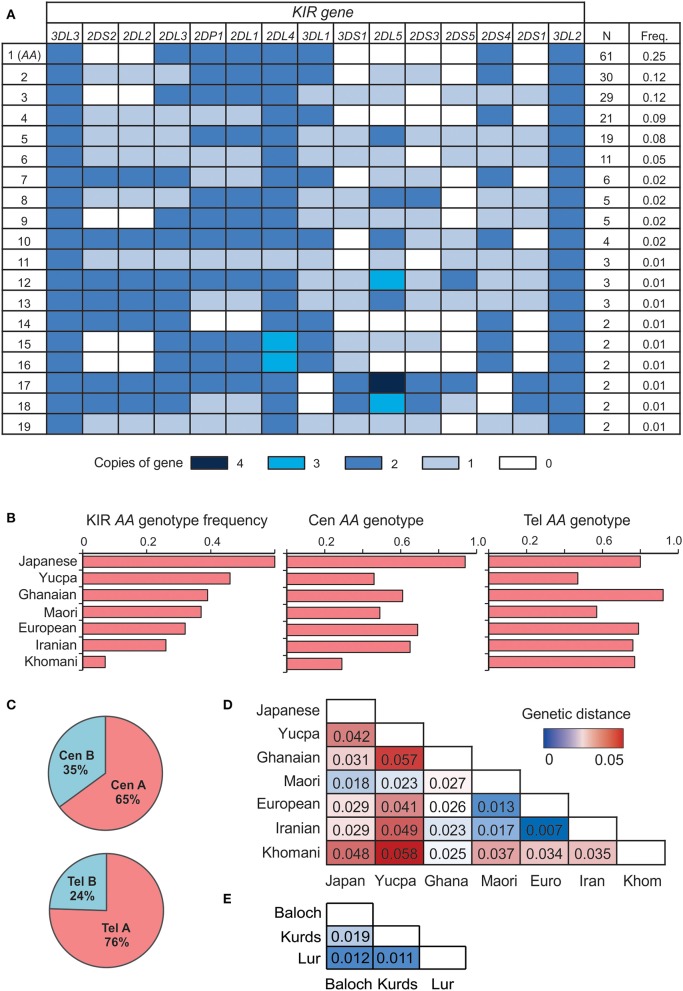
Iranian *KIR* genotypes resemble those of Europeans. **(A)** Shows *KIR* gene copy-number genotypes ordered by the total number observed across the five Iranian populations. Only genotypes present in more than one individual are shown. Colored boxes indicate the number of copies of the gene, as given in the key below. A white box indicates the gene is absent. **(B)** Frequency of the full *KIR AA* (left), *Cen AA* (center), and *Tel AA* (right) genotypes across seven representative world populations, ordered by frequency of full *KIR AA*. The populations are Japanese (*N* = 115), Yucpa Amerindians (*N* = 61), Ghanaians from West Africa (*N* = 131), Maori from New Zealand (*N* = 49), Europeans (*N* = 378), Iranians (*N* = 241), and Khomani from South Africa (*N* = 79). **(C)** Frequency of centromeric (Cen) and telomeric (Tel) *KIR A* and *B* haplotypes in the five Iranian populations combined. **(D)** Genetic distance between the combined Iranian population and the six representative, populations from **(B)**. *KIR* genes included are those genotyped in all the populations shown 2*DL1-4, 2DS1-5, 3DL1/S1, 3DL2*. **(E)** Genetic distance between the three Iranian populations.

We sequenced the *KIR* genes of 241 individuals from five populations, representing three groups of Iranians; the Lurs, Kurds and Baloch. The Lurs comprised individuals from the cities of Khoramabad, Lordegan and Yasuj. Only two *KIR* genes were present as two copies (2N) in every individual, *KIR3DL3* at the centromeric end of the *KIR* locus and *KIR3DL2* at the telomeric end (Figure 1A). Every *KIR* haplotype in this Iranian cohort is therefore flanked by these two framework genes. The third framework gene is *KIR2DL4* (9). Because eight individuals have only one copy of *KIR2DL4* and six have three copies, *KIR2DL4* is not present on every Iranian *KIR* haplotype (Figure 1A), but is likely duplicated on some haplotypes and deleted from others (45). The frequency of *KIR-A* haplotypes varies across populations (16, 46). In the combined study population, the frequency of *KIR AA* genotypes observed is 25% (Figure 1B). C*entromeric KIR-A* haplotypes are present at 65% and *telomeric KIR-A* haplotypes at 76% (Figure 1C). The frequency of *KIR A* haplotype homozygotes closely matches that of European populations (Figure 1B), as do genetic distance measurements calculated from the *KIR* genotype data (Figure 1D). In conclusion, the number and distribution of *KIR* gene content haplotypes in Iranians closely resemble those present in Europeans.

**Figure 2:**
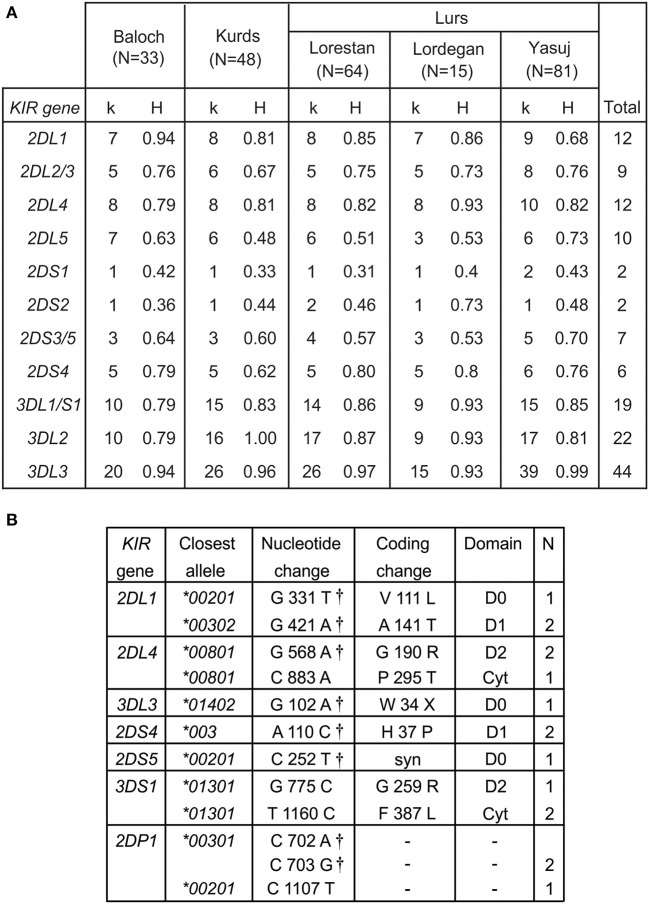
High *KIR* diversity and allele discovery in Iranians. **(A)** Shows the numbers of *KIR* alleles (k) and the heterozygosity (H) in each of the five Iranian populations. The total number observed is shown at the right. Gene absence is not included as an allele. **(B)** Shows the novel *KIR* alleles identified in this study. Columns from left to right are: the *KIR* gene, the closest known allele, the nucleotide change compared to the closest allele, the amino acid substitution caused by the nucleotide change, domain affected by the amino acid substitution (LP, leader peptide; D0–D1, Ig-like domains; TM, transmembrane domain) and the number observed. †-indicates identical allele observed by Wagner et al. (47).

We determined the *KIR* allele frequencies in the five Iranian populations. These data are shown in Supplementary Material A and summarized in Figure 2. The allele frequencies were consistent with Hardy-Weinberg equilibrium. Among the five populations, we identified 115 inhibitory KIR alleles and 18 activating KIR alleles (Figure 2A). Also present are 12 *KIR2DL4* alleles. Inhibitory KIR are highly polymorphic in Iranians, with 12 *KIR2DL1*, 9 *KIR2DL2/L3*, 10 *KIR2DL5*, 22 *KIR3DL2*, 18 *KIR3DL1*, and 44 *KIR3DL3* alleles observed in total. As in other populations, the activating KIR are less polymorphic than the inhibitory KIR, with 7 *KIR2DS4* alleles, 8 *KIR2DS3/5* alleles, two *KIR2DS1* and two *KIR2DS2* alleles and one *KIR3DS1* allele being seen (Figure 2A). In Iranians, *KIR3DL3* is the most polymorphic *KIR* gene, whereas *KIR2DS1* and *KIR2DS2* are the least variable. In the combined population analyzed, the most frequent allele for each of the inhibitory KIR are 2DL1^*^00302, 2DL2^*^00101, 2DL3^*^00101, 3DL1^*^00101, 3DL2^*^00101, and 3DL3^*^00301, respectively (Supplementary Material A). The most common KIR2DL4 and KIR2DS4 alleles are 2DL4^*^00801 and 2DS4^*^003, respectively. For the other activating KIR (KIR2DS1-3 and 2DS5) as well as KIR2DL5, the most frequent allele observed is absence of the gene (Supplementary Material A). We did not observe any statistically significant difference in frequency of any specific *KIR* allele between Kurs and Lurs. We identified 11 novel *KIR* alleles, eight being defined by amino acid substitutions, one by a synonymous substitution and two by substitutions in *KIR2DP1* pseudogene (Figure 2B). All these novel alleles were observed in one or two individuals (Figure 2B). Seven of them have sequences identical to ones reported recently in a survey of more than one million registered bone marrow donors (47).

**Figure 3:**
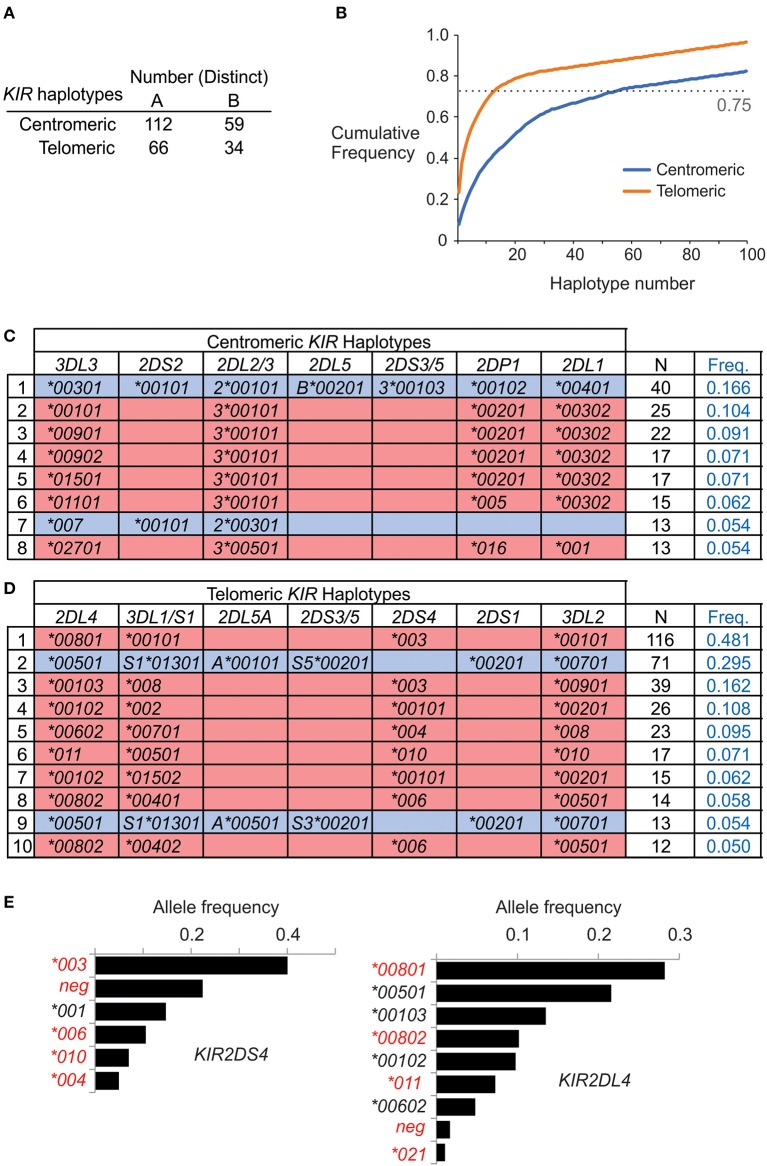
Centromeric *KIR* are more diverse than telomeric *KIR* in Iranians. **(A)** Shows the number of distinct haplotypes observed in the centromeric and telomeric *KIR* regions. **(B)** Shows the cumulative frequency of haplotypes observed in the centromeric and telomeric *KIR* regions. **(C,D)** Shows the allele composition of all **(C)** centromeric, and **(D)** telomeric *KIR* haplotypes identified in more than ten individuals in the combined Iranian population. *KIR A* haplotypes are shaded pink and *KIR B* haplotypes are blue. Empty boxes indicate gene absence. At the right is shown number observed and the frequency in the combined Iranian population (*N* = 241). All the observed haplotypes are given in Supplementary Material (E). Shown are the frequencies of *KIR2DL4* and *KIR2DS4* alleles in the combined Iranian population. Red text indicates alleles not expressed at cell surface. The allele frequencies for all *KIR* genes in each of the five populations are given in Supplementary Material.

On the basis of allele composition, we defined 170 centromeric and 94 telomeric *KIR* haplotypes (Supplementary Materials B,C). Thus, by distinct haplotype number, the centromeric region is twice as diverse as the telomeric region. Emphasizing this difference, the 55 most frequent centromeric haplotypes account for 75% of the total haplotypes, whereas only 13 telomeric haplotypes are sufficient to account for 75% of the telomeric haplotypes (Figures 3A,B). In conclusion, the centromeric *KIR* region of Iranian *KIR* haplotypes is far more diverse than the telomeric *KIR* region.

Although *KIR*-*A* haplotypes are more numerous and frequent than *KIR-B* haplotypes (Figure 1), the most frequent centromeric region and the second most frequent telomeric region haplotypes are *KIR-B* (Figures 3C,D). Together, these centromeric and telomeric segments encode only one inhibitory receptor specific for HLA class I (KIR2DL2^*^001). Also encoded are KIR2DL1^*^004, an attenuated receptor (23) and KIR3DL2^*^007, which has a mutation in the first ITIM of the cytoplasmic tail (48) and thus may not transmit an inhibitory signal. By contrast, the *KIR*-*B* haplotype encodes three functional activating receptors (KIR2DS1, 2DS2, and 3DS1) and a full-length KIR2DL4 (^*^005). This combination of common centromeric and telomeric *KIR-B* haplotypes can therefore provide the maximum number of activating KIR. The most frequent *KIR*-*A* haplotype encodes four inhibitory receptors specific for polymorphic HLA class I and carries KIR2DS4^*^003, which is not-expressed because of a 22 bp deletion in exon 5 (11). Indeed, the frequency of the *KIR2DS4 22 bp-del* variant in Iranians is 0.62, more than four times higher than the frequency of the full-length variant (Figure 3E). Consequently, almost all *KIR-A* haplotypes in Iranians encode four functional inhibitory receptors and no activating receptor specific for polymorphic HLA class I.

**Figure 4:**
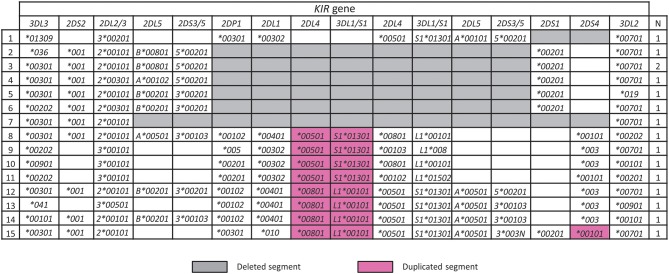
Rare structural variants of KIR haplotypes in Iranians. Shown are Iranian *KIR* haplotypes affected by a copy number variation. Gray and purple boxes highlight deleted or duplicated segments, respectively. “*N*” indicates the number observed.

A characteristic of the *KIR* locus is the occurrence of large-scale duplication or deletion events that encompass complete genes, which synergizes with allele variation to enhance KIR functional diversity (45, 49, 50). In the Iranian cohort, we identified seven *KIR* haplotypes having large deletions and eight with duplications (Figure 4). One of these haplotypes (number 1 in Figure 4) was not observed previously and is similar to the most frequent *KIR*-*B* haplotype in Iran, with the difference being that it lacks *KIR2DS1*. This haplotype could have been formed by homologous or looping out recombination (50). The remaining deletion haplotypes are similar to those observed worldwide including Africans, and the duplication haplotypes similar to those observed outside of Africa (45).

**Figure 5:**
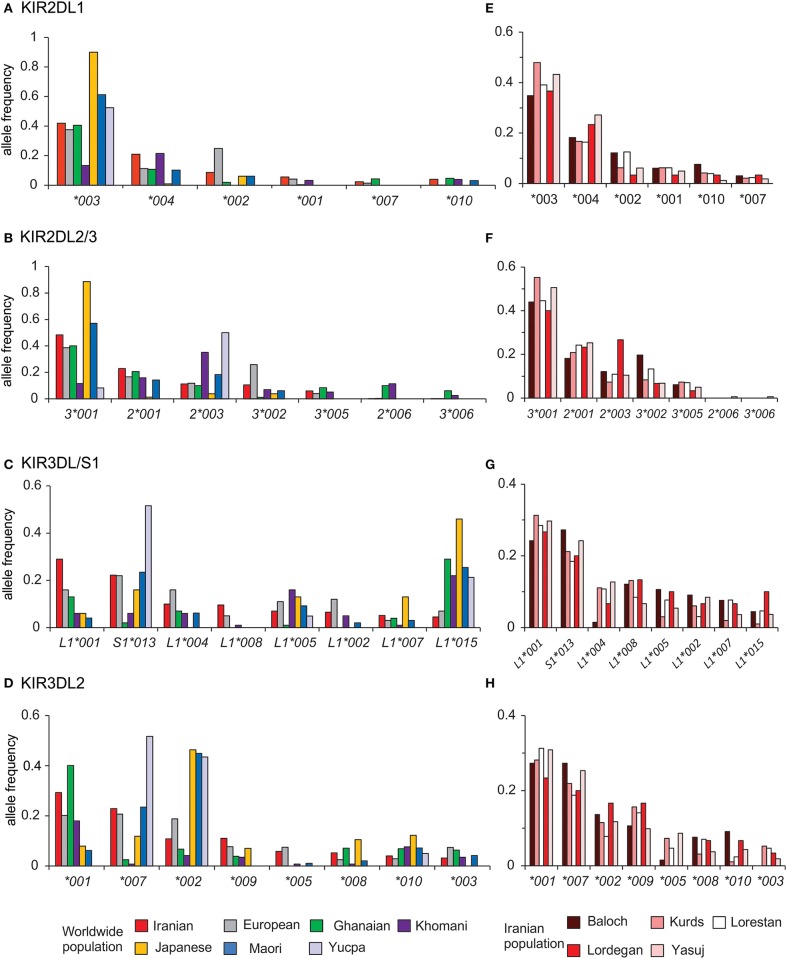
Telomeric *KIR* are more divergent than centromeric *KIR* across populations. Shows the alleles of **(A)** *KIR2DL1*, **(B)** *KIR2DL2*/3, **(C)** *KIR3DL1/S1*, and **(D)** *KIR3DL2* observed in Iran, and their frequencies in the combined Iranian population and six other populations representing major world groups (Left). **(E-H)** show the allele frequencies in the individual Iranian populations. The populations are Iranians (*N* = 241, comprised from Baloch *N* = 33, Kurds *N* = 48, Lorestan *N* = 64, Lordegan *N* = 15, and Yasuj *N* = 81), Ghanaians (*N* = 131), Khomani (*N* = 79), Maori (*N* = 49), Japanese (*N* = 115), Europeans (*N* = 378), and Yucpa (*N* = 61). The allele frequencies for all *KIR* genes in each of the five Iranian populations are given in Supplementary Material.

The interaction of HLA class I with inhibitory KIR contributes to NK cell education (51, 52). We analyzed the distribution of alleles for the four inhibitory KIR that are specific for HLA class I in Iranians and compared them with six other populations that represent the breadth of human genetic diversity (Figure 5). This analysis showed that in Iranians, Europeans, West Africans, and Japanese the most frequent *KIR2DL1* and *KIR2DL2/3* alleles are 2DL1^*^003 and 2DL3^*^001, respectively. The two populations that differ are relatively small indigenous populations (Figures 5A,B). By contrast, the *KIR3DL1/S1* and *KIR3DL2* alleles most frequent in Iranians are usually not the same as those most frequent in other populations (Figures 5C,D). This shows there is a greater worldwide divergence of KIR specific for HLA-A and -B than of KIR specific for HLA-C. Of particular note, Iranian populations have the highest frequency of 3DL1^*^001 (0.29) compared to the other six populations as well as to all other populations analyzed to date (53). Allele frequencies of the four inhibitory *KIR* specific for HLA class I are similar across the Iranian populations analyzed (Figures 5E–H). The one exception is the Baloch who have a low frequency of KIR3DL1^*^004, as well as KIR3DL2^*^003 and ^*^005, which are in strong linkage disequilibrium with 3DL1^*^004 (28, 44) (Supplementary Material C). The Baloch have one tenth the frequency of 3DL1^*^004 (0.015) than Kurd [0.114, χ2 *p* = 0.006; pc = 0.06(ns)], Lorestan [0.109, χ2 *p* = 0.006; pc = 0.06(ns)] and Yasuj [0.129, χ2 *p* = 0.003; pc = 0.03]. KIR3DL1^*^004 is retained in the cytoplasm and unable to bind HLA-Bw4+ HLA-A or -B on target cells (54).

**Figure 6:**
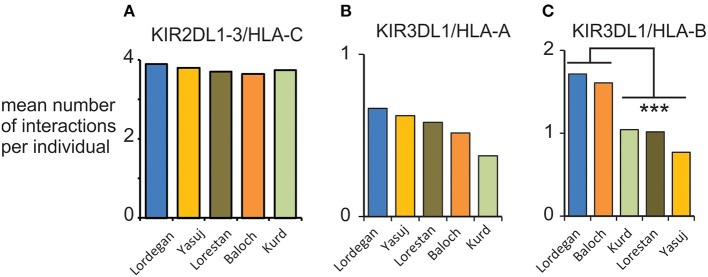
Interactions between KIR and HLA class I in five Iranian populations. **(A)** Shows the mean total number of viable interactions per individual of inhibitory KIR and HLA-C. **(B)** Shows the mean total number of viable interactions per individual of KIR3DL1 and HLA-A. **(C)** Shows the mean total number of viable interactions per individual of KIR3DL1 and HLA-B. ***p < 0.013, obtained from a two-tailed Mann-Whitney *U*-test.

We examined the compound genotypes of *KIR* and *HLA class I*, to determine the potential number of interactions between HLA class I ligands and inhibitory KIR. We observed a consistent mean of four viable interactions per individual of HLA-C with inhibitory KIR across the five Iranian populations (Figure 6A). This number is similar to the mean of 3.6 observed in Europeans (28). Although we observed no significant difference in the potential interactions of KIR3DL1 with HLA-A, the Baloch and Lordegan have more interactions of KIR3DL1 with HLA-B than the other three Iranian populations (Figures 6B,C). Despite the small numbers of individuals in these groups, the differences are statistically significant (Mann-Whitney *U*-test; *p* < 0.013). Contributing to these differences is the high frequency of HLA-B^*^51 in the Baloch (0.29; Supplementary Material D). This frequency is similar to the 0.28 observed in the Lordegan and considered the highest worldwide (34).

## Discussion

This study applied high-throughput, next-generation sequencing to define *KIR* polymorphism at high resolution in five Iranian populations. These comprise the Kurd and Lur populations from the Zagros Mountains in the west of Iran and the Baloch from the south-east. The Lur comprised three subpopulations; the Lorestan, Lordegan, and Yasuj. *HLA class I* allele distributions for four of these populations were reported previously (34) whereas those for the Baloch are described here. When we compared the *KIR* allele frequencies of Iranians to those representing African, Asian, European, Oceanian, and South American populations, we found that the Iranian groups we studied are particularly similar to Europeans. This finding is consistent with ancient DNA analysis, which revealed that Iranians and Europeans both originate from an Indo-Europeans steppe ancestor population (55).

Despite their overall similarity, the Iranians we studied differ from Europeans in their frequencies of KIR3DL1^*^001, a high-expressing inhibitory receptor specific for the Bw4 epitope of subsets of HLA-A and -B allotypes. Iranians have KIR3DL1^*^001 frequencies that are twice those in Europeans and are the highest worldwide. Iran has the highest frequency of HLA-B^*^51 in the world (34), and this is also the case for the Baloch. HLA-B^*^51 has the Bw4 epitope and thus educates KIR3DL1^+^ NK cells to detect any loss in Bw4^+^ HLA-A or -B expression (35, 56, 57). HLA-B^*^51 is associated with Behçet disease, a chronic, multi-system autoimmune condition that has substantially higher incidence in Iran (80/100,000) than Europe (<1/100,000) (58, 59). We recently showed that high-expressing allotypes of KIR3DL1, including 3DL1^*^001, can protect from Behçet disease (60). It is unlikely that KIR3DL1^*^001 and HLA-B^*^51 rose to high frequency in Iran to protect specifically from an autoimmune disease, but this combination of HLA and KIR could also protect against specific infectious diseases (7). Examples include tuberculosis and hepatitis, which are both prevalent in Balochistan (61–63). Amongst Iranians, the Baloch and Lordegan have the highest number of viable interactions between KIR3DL1 and Bw4^+^HLA. Contributing to this high occurrence in the Baloch, are high frequencies of KIR3DL1^*^001 and HLA-B^*^51 and a low frequency of KIR3DL1^*^004. That both the *KIR3DL2* alleles linked to KIR3DL1^*^004 are also reduced in frequency suggests that KIR3DL1^*^004 has been specifically targeted by negative selection in the Baloch, rather than another *KIR* allele in linkage disequilibrium with KIR3DL1^*^004.

*KIR-A* and *-B* haplotypes are present in all human populations, where they are maintained by balancing selection, likely because *KIR-A* haplotypes favor infection control, particularly viral infections, and *KIR-B* haplotypes favor successful fetal implantation (1, 16). Accordingly, *KIR-A* haplotypes express all possible inhibitory receptors specific for HLA class I, whereas *KIR-B* haplotypes express fewer inhibitory receptors but more activating receptors. Thus, whereas the inhibitory KIR help prime NK cells to be able to be responsive to HLA class I loss during infection, the activating KIR can both promote fetal trophoblast invasion and recognize specific pathogen-derived peptides to control certain infections (64, 65). In Iranian populations the differences between the *KIR-A* and *KIR-B* haplotypes is extreme. The common *KIR-A* haplotypes express no activating KIR, due to the high frequency of the truncated KIR2DS4 variant, and the common *KIR-B* haplotypes provide the maximum number of activating receptors. In this regard, the *KIR-B* haplotypes differ considerably from those of Africans and were likely obtained since the out of Africa migration, through adaptive introgression with ancient humans (66).

In summary, we describe the *KIR* locus at allelic resolution in Iranian populations and place it in the context of the HLA ligands recognized by KIR. Iran is a culturally diverse country and the ethnic groups we have studied comprise ~15% of the population (67). Because substantial *KIR* gene content diversity is observed across Iran (68–71), it will be of interest in future studies to compare our results with other Iranian populations including Persians and Azeris. The allele and haplotype distributions described here will provide a baseline for future studies of disease association and transplantation matching in this important region of the world.

## Data Availability Statement

All relevant data are included in the article/Supplementary Material. Other raw data supporting the conclusions of this article will be made available by the authors, without undue reservation, to any qualified researcher.

## Ethics Statement

The studies involving human participants were reviewed and approved by the Medical Research Ethics Committee of Shiraz University of Medical Sciences. The patients/participants provided their written informed consent to participate in this study.

## Author Contributions

PP, EA, AG, and PN conceived and designed experiments. EA, NN-G, and ST performed lab experiments. CA, PN, JAT, LG, NN-G, and ST analyzed data. AG, PP, WM, JH, JT, and LM provided materials. CA, PN, and PP wrote the paper. All authors approved the final submitted version.

### Conflict of Interest

The authors declare that the research was conducted in the absence of any commercial or financial relationships that could be construed as a potential conflict of interest.

## Supplementary Material

Supplementary Material (Spreadsheet) **(A)** Shown are the allele frequencies for the 13 *KIR* genes in five Iranian populations, and the combined frequency is shown at the right. **(B)** Shown are the *centromeric KIR* haplotypes identified in five Iranian populations. The combined frequency is shown at the right. *KIR A* haplotypes are shaded in red, *KIR B* haplotypes are shaded in blue. **(C)** Shown are the *telomeric KIR* haplotypes identified in five Iranian populations. The combined frequency is shown at the right. *KIR A* haplotypes are shaded in red, *KIR B* haplotypes are shaded in blue. Yellow shading indicates novel *KIR* alleles identified in this study, which are described in Figure 2. **(D)** Shows the *HLA class I* allele frequencies observed in the Baloch population.

## References

[1] Parham P Moffett A. Variable NK cell receptors and their MHC class I ligands in immunity, reproduction and human evolution. Nat Rev Immunol. (2013) 13:133–44. 10.1038/nri3370 23334245 PMC3956658

[2] Vance RE Eichberg MJ Portnoy DA Raule D. Listening to each other: infectious disease and cancer immunology. Sci Immunol. (2017) 2:eaai9339. 10.1126/sciimmunol.aai9339 28783669 PMC5927821

[3] Long EO Kim HS Liu D Peterson ME Rajagopalan S. Controlling natural killer cell responses: integration of signals for activation and inhibition. Annu Rev Immunol. (2013) 31:227–58. 10.1146/annurev-immunol-020711-075005 23516982 PMC3868343

[4] Lam VC Lanier LL. NK cells in host responses to viral infections. Curr Opin Immunol. (2017) 44:43–51. 10.1016/j.coi.2016.11.003 27984782 PMC5451301

[5] Stewart CA Vivier E Colonna M. Strategies of natural killer cell recognition and signaling. Curr Top Microbiol Immunol. (2006) 298:1–21. 10.1007/3-540-27743-9_1 16329183

[6] Moretta L Pietra G Montaldo E Vacca P Pende D Falco M. Human NK cells: from surface receptors to the therapy of leukemias and solid tumors. Front Immunol. (2014) 5:87. 10.3389/fimmu.2014.00087 24639677 PMC3945935

[7] Bashirova AA Martin MP McVicar DW Carrington M. The killer immunoglobulin-like receptor gene cluster: tuning the genome for defense. Annu Rev Genomics Hum Genet. (2006) 7:277–300. 10.1146/annurev.genom.7.080505.115726 16824023

[8] Locatelli F Pende D Falco M Della Chiesa M Moretta A Moretta L. NK cells mediate a crucial graft-versus-leukemia effect in haploidentical-HSCT to cure high-risk acute leukemia. Trends Immunol. (2018) 39:577–90. 10.1016/j.it.2018.04.009 29793748

[9] Wilson MJ Torkar M Haude A Milne S Jones T Sheer D. Plasticity in the organization and sequences of human KIR/ILT gene families. Proc Natl Acad Sci USA. (2000) 97:4778–83. 10.1073/pnas.080588597 10781084 PMC18309

[10] Uhrberg M Valiante NM Shum BP Shilling H Lienert-Weidenbach K Corliss B. Human diversity in killer cell inhibitory receptor genes. Immunity. (1997) 7:753–63. 10.1016/S1074-7613(00)80394-5 9430221

[11] Hsu KC Liu XR Selvakumar A Mickelson E O'Reilly R Dupont B. Killer Ig-like receptor haplotype analysis by gene content: evidence for genomic diversity with a minimum of six basic framework haplotypes, each with multiple subsets. J Immunol. (2002) 169:5118–29. 10.4049/jimmunol.169.9.5118 12391228

[12] Marsh SG Parham P Dupont B Geraghty DE Trowsdale J Middleton D. Killer-cell immunoglobulin-like receptor (KIR) nomenclature report, 2002. Tissue Antigens. (2003) 62:79–86. 10.1034/j.1399-0039.2003.00072.x 12859599

[13] Kikuchi-Maki A Catina TL Campbell KS. Cutting edge: KIR2DL4 transduces signals into human NK cells through association with the Fc receptor gamma protein. J Immunol. (2005) 174:3859–63. 10.4049/jimmunol.174.7.3859 15778339

[14] Miah SM Hughes TL Campbell KS. KIR2DL4 differentially signals downstream functions in human NK cells through distinct structural modules. J Immunol. (2008) 180:2922–32. 10.4049/jimmunol.180.5.2922 18292514

[15] Rajagopalan S Long EO. KIR2DL4 (CD158d): an activation receptor for HLA-G. Front Immunol. (2012) 3:258. 10.3389/fimmu.2012.00258 22934097 PMC3422731

[16] Guethlein LA Norman PJ Hilton HH Parham P. Co-evolution of MHC class I and variable NK cell receptors in placental mammals. Immunol Rev. (2015) 267:259–82. 10.1111/imr.12326 26284483 PMC4587382

[17] Pyo CW Guethlein LA Vu Q Wang R Abi-Rached L Norman PJ. Different patterns of evolution in the centromeric and telomeric regions of group A and B haplotypes of the human killer cell Ig-like receptor locus. PLoS ONE. (2010) 5:e15115. 10.1371/journal.pone.0015115 21206914 PMC3012066

[18] Cooley S Weisdorf DJ Guethlein LA Klein J Wang T Le CT. Donor selection for natural killer cell receptor genes leads to superior survival after unrelated transplantation for acute myelogenous leukemia. Blood. (2010) 116:2411–9. 10.1182/blood-2010-05-283051 20581313 PMC2953880

[19] Oevermann L Michaelis SU Mezger M Lang P Toporski J Bertaina A. KIR B haplotype donors confer a reduced risk for relapse after haploidentical transplantation in children with all. Blood. (2014) 124:2744–7. 10.1182/blood-2014-03-565069 25115891 PMC4208288

[20] Winter CC Gumperz JE Parham P Long EO Wagtmann N. Direct binding and functional transfer of NK cell inhibitory receptors reveal novel patterns of HLA-C allotype recognition. J Immunol. (1998) 161:571–7. 9670929

[21] Pando MJ Gardiner CM Gleimer M McQueen KL Parham P. The protein made from a common allele of KIR3DL1 (3DL1^*^004) is poorly expressed at cell surfaces due to substitution at positions 86 in Ig domain 0 182 in Ig domain 1. J Immunol. (2003) 171:6640–9. 10.4049/jimmunol.171.12.6640 14662867

[22] Moesta AK Norman PJ Yawata M Yawata N Gleimer M Parham P. Synergistic polymorphism at two positions distal to the ligand-binding site makes KIR2DL2 a stronger receptor for HLA-C than KIR2DL3. J Immunol. (2008) 180:3969–79. 10.4049/jimmunol.180.6.3969 18322206

[23] Bari R Bell T Leung WH Vong QP Chan WK Das Gupta N. Significant functional heterogeneity among KIR2DL1 alleles and a pivotal role of arginine 245. Blood. (2009) 114:5182–90. 10.1182/blood-2009-07-231977 19828694 PMC2792213

[24] VandenBussche CJ Mulrooney TJ Frazier WR Dakshanamurthy S Hurley CK. Dramatically reduced surface expression of NK cell receptor KIR2DS3 is attributed to multiple residues throughout the molecule. Genes Immun. (2009) 10:162–73. 10.1038/gene.2008.91 19005473 PMC3487464

[25] Hilton HG Guethlein LA Goyos A Nemat-Gorgani N Bushnell D Norman PJ. Polymorphic HLA-C receptors balance the functional characteristics of KIR haplotypes. J Immunol. (2015) 195:3160–70. 10.4049/jimmunol.1501358 26311903 PMC4575877

[26] Gendzekhadze K Norman PJ Abi-Rached L Graef T Moesta A Layrisse Z. Co-evolution of KIR2DL3 with HLA-C in a human population retaining minimal essential diversity of KIR and HLA class I ligands. Proc Natl Acad Sci USA. (2009) 106:18692–7. 10.1073/pnas.0906051106 19837691 PMC2774017

[27] Norman PJ Hollenbach JA Nemat-Gorgani N Guethlein LA Hilton HG Pando MJ. Co-evolution of human leukocyte antigen (HLA) class I ligands with killer-cell immunoglobulin-like receptors (KIR) in a genetically diverse population of sub-Saharan Africans. PLoS Genet. (2013) 9:e1003938. 10.1371/journal.pgen.1003938 24204327 PMC3814319

[28] Nemat-Gorgani N Hilton HG Henn BM Lin M Gignoux CR Myrick JW. Different selected mechanisms attenuated the inhibitory interaction of KIR2DL1 with C2(+) HLA-C in two indigenous human populations in Southern Africa. J Immunol. (2018) 200:2640–55. 10.4049/jimmunol.1701780 29549179 PMC5990024

[29] Nemat-Gorgani N Guethlein LA Henn BM Norberg S Chiaroni J Sikora M. Diversity of KIR, HLA class I, their interactions in seven populations of sub-Saharan Africans. J Immunol. (2019) 202:2636–47. 10.4049/jimmunol.1801586 30918042 PMC6690726

[30] Middleton D Meenagh A Gourraud PA. KIR haplotype content at the allele level in 77 Northern Irish families. Immunogenetics. (2007) 59:145–58. 10.1007/s00251-006-0181-7 17200871

[31] Vierra-Green C Roe D Hou L Hurley CK Rajalingam R Reed E. Allele-level haplotype frequencies and pairwise linkage disequilibrium for 14 KIR loci in 506 European-American individuals. PLoS ONE. (2012) 7:e47491. 10.1371/journal.pone.0047491 23139747 PMC3489906

[32] Yawata M Yawata N Draghi M Little AM Partheniou F Parham P. Roles for HLA and KIR polymorphisms in natural killer cell repertoire selection and modulation of effector function. J Exp Med. (2006) 203:633–45. 10.1084/jem.20051884 16533882 PMC2118260

[33] Nemat-Gorgani N Edinur HA Hollenbach JA Traherne J Dunn PP Chambers GK KIR diversity in Maori and Polynesians: populations in which HLA-B is not a significant KIR ligand. Immunogenetics. (2014) 66:597–611. 10.1007/s00251-014-0794-1 25139336 PMC4198482

[34] Ashouri E Norman PJ Guethlein LA Han A Nemat-Gorgani N Norberg SJ. HLA class I variation in Iranian Lur and Kurd populations: high haplotype and allotype diversity with an abundance of KIR ligands. HLA. (2016) 88:87–99. 10.1111/tan.12852 27558013 PMC5063635

[35] Cella M Longo A Ferrara GB Strominger JL Colonna M. NK3-specific natural killer cells are selectively inhibited by Bw4-positive HLA alleles with isoleucine 80. J Exp Med. (1994) 180:1235–42. 10.1084/jem.180.4.1235 7931060 PMC2191670

[36] Saunders PM Pymm P Pietra G Hughes VA Hitchen C O'Connor GM. Killer cell immunoglobulin-like receptor 3DL1 polymorphism defines distinct hierarchies of HLA class I recognition. J Exp Med. (2016) 213:791–807. 10.1084/jem.20152023 27045007 PMC4854737

[37] Norman PJ Hollenbach JA Nemat-Gorgani N Marin WM Norberg SJ Ashouri E. Defining KIR and HLA class I genotypes at highest resolution via high-throughput sequencing. Am J Hum Genet. (2016) 99:375–91. 10.1016/j.ajhg.2016.06.023 27486779 PMC4974113

[38] Langmead B Salzberg S L. Fast gapped-read alignment with Bowtie 2. Nat Methods. (2012) 9:357–9. 10.1038/nmeth.1923 22388286 PMC3322381

[39] Chevreux B Pfisterer T Drescher B Driesel AJ Muller W Wetter T. Using the miraEST assembler for reliable and automated mRNA transcript assembly and SNP detection in sequenced ESTs. Genome Res. (2004) 14:1147–59. 10.1101/gr.1917404 15140833 PMC419793

[40] Bonfield JK Whitwham A. Gap5–editing the billion fragment sequence assembly. Bioinformatics. (2010) 26:1699–703. 10.1093/bioinformatics/btq268 20513662 PMC2894512

[41] Thorvaldsdottir H Robinson JT Mesirov JP. Integrative genomics viewer (IGV): high-performance genomics data visualization and exploration. Brief Bioinformatics. (2013) 14:178–92. 10.1093/bib/bbs017 22517427 PMC3603213

[42] Stephens M Donnelly P. A comparison of bayesian methods for haplotype reconstruction from population genotype data. Am J Hum Genet. (2003) 73:1162–9. 10.1086/379378 14574645 PMC1180495

[43] Cavalli-Sforza LL Edwards AW. Phylogenetic analysis. models and estimation procedures. Am J Hum Genet. (1967) 19(3 Pt 1):233–57. 10.2307/2406616 6026583 PMC1706274

[44] Misra MK Augusto DG Martin GM Nemat-Gorgani N Sauter J Hofmann JA. Report from the killer-cell immunoglobulin-like receptors (KIR) component of the 17th international hla and immunogenetics workshop. Hum Immunol. (2018) 79:825–33. 10.1016/j.humimm.2018.10.003 30321631 PMC6322681

[45] Norman PJ Abi-Rached L Gendzekhadze K Hammond JA Moesta A Sharma D. Meiotic recombination generates rich diversity in NK cell receptor genes, alleles, and haplotypes. Genome Res. (2009) 19:757–69. 10.1101/gr.085738.108 19411600 PMC2675964

[46] Hollenbach JA Nocedal I Ladner MB Single RM Trachtenberg EA. Killer cell immunoglobulin-like receptor (KIR) gene content variation in the HGDP-CEPH populations. Immunogenetics. (2012) 64:719–37. 10.1007/s00251-012-0629-x 22752190 PMC3438391

[47] Wagner I Schefzyk D Pruschke J Schofl G Schone B Gruber N. Allele-level KIR genotyping of more than a million samples: workflow, algorithm, and observations. Front Immunol. (2018) 9:2843. 10.3389/fimmu.2018.02843 30564239 PMC6288436

[48] Robinson J Halliwell JA Hayhurst JD Flicek P Parham P Marsh SG. The IPD and IMGT/HLA database: allele variant databases. Nucleic Acids Res. (2015) 43:D423–31. 10.1093/nar/gku1161 25414341 PMC4383959

[49] Martin MP Bashirova A Traherne J Trowsdale J Carrington M. Cutting edge: expansion of the KIR locus by unequal crossing over. J Immunol. (2003) 171:2192–5. 10.4049/jimmunol.171.5.2192 12928362

[50] Traherne JA Martin M Ward R Ohashi M Pellett F Gladman D. Mechanisms of copy number variation and hybrid gene formation in the KIR immune gene complex. Hum Mol Genet. (2010) 19:737–51. 10.1093/hmg/ddp538 19959527 PMC2816608

[51] Anfossi N Andre P Guia S Falk CS Roetynck S Stewart CA. Human NK cell education by inhibitory receptors for MHC class I. Immunity. (2006) 25:331–42. 10.1016/j.immuni.2006.06.013 16901727

[52] Goodridge JP Jacobs B Saetersmoen ML Clement D Hammer Q Clancy T. Remodeling of secretory lysosomes during education tunes functional potential in NK cells. Nat Commun. (2019) 10:514. 10.1038/s41467-019-08384-x 30705279 PMC6355880

[53] Norman PJ Abi-Rached L Gendzekhadze K Korbel D Gleimer M Rowley D. Unusual selection on the KIR3DL1/S1 natural killer cell receptor in Africans. Nat Genet. (2007) 39:1092–9. 10.1038/ng2111 17694054

[54] Taner SB Pando MJ Roberts A Schellekens J Marsh S Malmberg KJ. Interactions of NK cell receptor KIR3DL1^*^004 with chaperones and conformation-specific antibody reveal a functional folded state as well as predominant intracellular retention. J Immunol. (2011) 186:62–72. 10.4049/jimmunol.0903657 21115737 PMC3129036

[55] Broushaki F Thomas MG Link V Lopez S van Dorp L Kirsanow K. Early neolithic genomes from the eastern Fertile Crescent. Science. (2016) 353:499–503. 10.1126/science.aaf7943 27417496 PMC5113750

[56] Gumperz JE Litwin V Phillips JH Lanier LL Parham P. The Bw4 public epitope of HLA-B molecules confers reactivity with natural killer cell clones that express NKB1, a putative HLA receptor. J Exp Med. (1995) 181:1133–44. 10.1084/jem.181.3.1133 7532677 PMC2191933

[57] Carr WH Pando MJ Parham P. KIR3DL1 polymorphisms that affect NK cell inhibition by HLA-Bw4 ligand. J Immunol. (2005) 175:5222–9. 10.4049/jimmunol.175.8.5222 16210627

[58] Ohno S Aoki K Sugiura S Nakayama E Itakura K Aizawa M. Letter: HL-A5 and Behcet's disease. Lancet. (1973) 2:1383–4. 10.1016/S0140-6736(73)93343-6 4128069

[59] Verity DH Wallace GR Vaughan RW Stanford M. Behcet's disease: from Hippocrates to the third millennium. Br J Ophthalmol. (2003) 87:1175–83. 10.1136/bjo.87.9.1175 12928293 PMC1771837

[60] Petrushkin H Norman PJ Lougee E Parham P Wallace G Stanford MR. KIR3DL1/S1 allotypes contribute differentially to the development of behcet disease. J Immunol. (2019) 203:1629–35. 10.4049/jimmunol.1801178 31405953 PMC6731450

[61] Sheikh NS Sheikh AS Sheikh AA Yahya S Rafi-U-Shan Lateef M. Sero-prevalence of hepatitis B virus infection in Balochistan Province of Pakistan. Saudi J Gastroenterol. (2011) 17:180–4. 10.4103/1319-3767.80380 21546720 PMC3122087

[62] Rahimi Foroushani A Farzianpour F Tavana A Rasouli J Hosseini S. The 10-year trend of TB rate in West Azerbaijan Province, Iran from 2001 to 2010. Iran J Public Health. (2014) 43:778–86. 26110148 PMC4475596

[63] WHO Global Tuberculosis Report 2019. Geneva: World Health Organization (2019).

[64] Mbiribindi B Mukherjee S Wellington D Das J Khakoo SI Spatial clustering of receptors and signaling molecules regulates NK cell response to peptide repertoire changes. Front Immunol. (2019) 10:605 10.3389/fimmu.2019.02370 31024524 PMC6460049

[65] Sim MJW Rajagopalan S Altmann DM Boyton RJ Sun PD Long EO. Human NK cell receptor KIR2DS4 detects a conserved bacterial epitope presented by HLA-C. Proc Natl Acad Sci USA. (2019) 116:201903781. 10.1073/pnas.1903781116 31138701 PMC6601252

[66] Abi-Rached L Jobin MJ Kulkarni S McWhinnie A Dalva K Gragert L. The shaping of modern human immune systems by multiregional admixture with archaic humans. Science. (2011) 334:89–94. 10.1126/science.1209202 21868630 PMC3677943

[67] Mehrjoo Z Fattahi Z Beheshtian M Mohseni M Poustchi H Ardalani F. Distinct genetic variation and heterogeneity of the Iranian population. PLoS Genet. (2019) 15:e1008385. 10.1371/journal.pgen.1008385 31550250 PMC6759149

[68] Ashouri E Farjadian S Reed EF Ghaderi A Rajalingam R. KIR gene content diversity in four Iranian populations. Immunogenetics. (2009) 61:483–92. 10.1007/s00251-009-0378-7 19521696 PMC2706385

[69] Tajik N Shahsavar F Mousavi T Radjabzadeh MF. Distribution of KIR genes in the Iranian population. Tissue Antigens. (2009) 74:22–31. 10.1111/j.1399-0039.2009.01263.x 19392787

[70] Hiby SE Ashrafian-Bonab M Farrell L Single RM Balloux F Carrington M. Distribution of killer cell immunoglobulin-like receptors (KIR) and their HLA-C ligands in two Iranian populations. Immunogenetics. (2010) 62:65–73. 10.1007/s00251-009-0408-5 19936734 PMC2814031

[71] Solgi G Ghafari H Ashouri E Alimoghdam K Rajalingam R Amirzargar A. (2011). Comparison of KIR gene content profiles revealed a difference between northern and southern Persians in the distribution of KIR2DS5 and its linked loci. Hum Immunol. 72:1079–83. 10.1016/j.humimm.2011.08.002 21867738

